# An Energy-Based Unified Approach to Predict the Low-Cycle Fatigue Life of Type 316L Stainless Steel under Various Temperatures and Strain-Rates

**DOI:** 10.3390/ma12071090

**Published:** 2019-04-02

**Authors:** Nae Hyung Tak, Jung-Seok Kim, Jae-Yong Lim

**Affiliations:** 1 Division of Industrial Metrology, Korea Research Institute of Standards and Science, Daejeon 34113, Korea; nhtak@kriss.re.kr; 2 Autonomous Vehicles Research Team, New Transportation Innovative Research Center, Korea Railroad Research Institute, Uiwang-si 16105, Korea; jskim@krri.re.kr; 3 Department of Safety Engineering, Seoul National University of Science and Technology, Seoul 01811, Korea

**Keywords:** type 316L stainless steel, low-cycle fatigue, fatigue life prediction, plastic strain energy density, elevated temperature

## Abstract

An energy-based low-cycle fatigue model was proposed for applications at a range of temperatures. An existing model was extended to the integrated approach, incorporating the simultaneous effects of strain rate and temperature. A favored material at high temperature, type 316L stainless steel, was selected in this study and its material characteristics were investigated. Tensile tests and low-cycle fatigue tests were performed using several strain rates at a temperature ranging from room temperature to 650 °C. Material properties were obtained in terms of temperature using the displacement-controlled tensile tests and further material response were investigated using strain-controlled tensile tests. Consequently, no pronounced reduction in strengths occurred at temperatures between 300 and 550 °C, and a negative strain rate response was observed in the temperature range. Based on the low-cycle fatigue tests by varying strain rates and temperature, it was found that a normalized plastic strain energy density and a strain-rate modified cycle were successfully correlated. The accuracy of the model was discussed by comparing between predicted and experimental lives.

## 1. Introduction

Reliable lifetime assessment is of primary concern in structures or mechanical components, most importantly in those whose failure results in catastrophic disaster. Because inaccurate or non-conservative estimations can cause significant losses in human lives and properties, the accuracy of predicted lives must be assured in the design stage and in the remaining useful lifetime assessment of the structures. Accuracy can be achieved on an integral basis of extensive explorations into material response, of external loading analysis, and of guaranteed life prediction models [1,2,3].

Many mechanical components in power facilities or nuclear reactors must withstand repeated loading at elevated temperatures and low-cycle fatigue (LCF) is considered as a predominant failure mechanism. For example, turbine blades, disks, and boiler components in plants are subjected to LCF at elevated temperatures. In those applications, the mechanical components can experience a wide range of operating temperatures. Specifically, the components are subject to temperature gradient induced cyclic thermal stresses owing to startups and shutdowns or the temperature change of a coolant that is either a liquid, such as sodium, or a gas, such as helium, that flows in the pipe of a cooling system [4,5]. Moreover, when exposed to a temperature greater than the creep-activation temperature, materials of which the components are made can experience complicated microstructural changes, resulting in appearance of various failure mechanisms. Thus, fatigue life prediction at elevated temperature can be a challenging problem.

A long history of studies exists to discover the effects associated with LCF behavior at elevated temperatures: Temperature, temperature cycling, strain rate, hold time, load sequence, etc. [6,7,8,9,10]. Each previous study concentrated on identifying and quantifying the individual effects on fatigue life. However, a more convincing lifetime calculation at the design stage and the remaining useful lifetime assessments requires a more inclusive approach that embraces a few of the above-mentioned effects because those are involved simultaneously. 

Two representative parameters describe the LCF characteristics: Plastic strain amplitude and plastic strain energy density. For example, the Coffin-Manson model [11] assumes a linear relationship between the plastic strain amplitude and fatigue life in the logarithmic coordinate and the Morrow model [12] relates a plastic strain energy density and fatigue life in the logarithmic coordinate. Subsequently, the strain rate effect on fatigue life was incorporated by developing the frequency-modified parameters in the frequency-modified Coffin-Manson model [13] and the frequency-modified energy model [14], respectively. The Coffin-Manson model and the Morrow energy model can predict well under the isothermal conditions, but they cannot explain the effect of strain rate on fatigue life. To consider the effect of strain rate on fatigue life, the frequency-modified Coffin-Manson model or the frequency-modified energy model should be used. Even though these two models can explain well the effects of strain rate and strain amplitude on fatigue life, they cannot account for the effect of temperature. They only represent how the material constants change with respect to the test temperatures. Therefore, it is better to improve and modify the existing models, including the Coffin-Manson model and the Morrow model mentioned above [11,12,13,14], to account for the temperature dependency of fatigue life.

In this study, type 316L stainless steel is selected to propose a phenomenological energy-based life prediction model incorporating strain rate and temperature effects. The material has been favored for power facilities or nuclear fusion reactors for its resistance at high temperatures. In this regard, tensile tests and strain-controlled LCF tests were conducted against various temperatures and strain rates. The temperatures ranges from room temperature (RT) to 650 °C, and the strain rate is varied from 1 × 10^−5^ to 1 × 10^−2^ s^−1^ to investigate the influence of strain rate and temperature on the material behavior and to measure the mechanical properties. Subsequently, the capabilities and validity of the developed model are discussed.

## 2. Materials and Methods 

### 2.1. Material and Specimen

**Figure 1:**
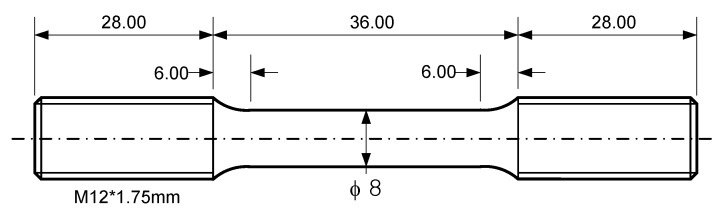
Specimen for tensile and low-cycle fatigue (LCF) test (dimensions in mm).

All specimens were made from an extruded 316L stainless steel bar of diameter 16 mm that was solution-treated at 1100 °C for 40 min and subsequently cold-drawn by 17% cold work (%CW). The chemical composition of the steel is shown in Table 1 and average grain size determined by linear intercept method was 53 μm. A type of specimen designed by ASTM code E606-92 [15] was employed for tensile tests and LCF tests as shown in Figure 1, and the specimen surface was polished along the longitudinal direction with emery paper up to 13 μm to remove surface defects.

### 2.2. Test Equipment

A closed-loop servo-hydraulic test system with a 5-ton capacity manufactured by MTS (Eden Prairie, MN, USA) was used to perform the tensile tests and LCF tests. A resistance type furnace that can control the ambient temperature with the variation of ±1 °C at steady state was used for temperature control. A specimen was fixed on the test machine using a hydraulic grip system. A high temperature extensometer manufactured by MTS (Model No.: 632-13F-20, guage length: 25 mm) was used to perform the strain control tests and the displacement signal, load signal, and strain signal were recorded for each cycle during the test.

### 2.3. Tesile Test

Tensile tests were performed in two different control manners: Displacement-control and strain-control. The tests were primarily performed using displacement-controlled tensile tests to obtain the mechanical properties depending on temperature. Additionally, strain-controlled tests, when further investigations were required, were subsequently performed to capture a particular mechanical response in the considered range of strain rate (1 × 10^−3^, 1 × 10^−4^ or 1 × 10^−5^ s^−1^). 

In the displacement-control tensile tests, the actuator is displaced at 2 mm/min under isothermal condition ranging from RT to 650 °C. Prior to loading, the material to be tested was metallurgically stabilized by maintaining the specimen at a test temperature for an hour. From the results, tensile properties such as *E*, σ_y_, σ_u_, %*EL* (percent elongation) were extracted.

For the strain-controlled tensile tests, specimens were prepared similarly as in the displacement-control test. Subsequently, they were stretched with a strain rate controlled by an extensometer. Because the extensometer used in the tests has a limited travel length with −5% ~ +10%, tensile tests using strain control were stopped at 5% strain value and stress relaxation tests were proceeded by maintaining this strain value. 

### 2.4. LCF Test

In order to investigate the effects of temperature and strain amplitude on fatigue life, LCF tests were performed using strain control. All tests were performed in air under a fully reversed total strain control mode employing a triangular waveform. A specimen was maintained at the test temperature for an hour before the fatigue test started while unloaded. 

First, extensive fatigue tests were performed to investigate the fatigue characteristics over a wide range of temperature, from RT to 650 °C. A fully reversed strain cycle using a strain rate of 1×10^−3^ s^−1^ was imposed under a carefully controlled temperature environment, and the considered strain amplitudes were 0.3%, 0.4%, 0.5%, 0.6%, 0.7%, 0.8% to construct a fatigue life curve for a given temperature. Likewise, the LCF tests were repeated to obtain the fatigue characteristics at other temperatures. Next, investigations to observe the effect of strain rate on fatigue life were performed. Here, the strain rates were varied with a fixed strain amplitude of 0.5%.

In this study, the fatigue life was defined as the 70% load drop point of a stabilized load amplitude at N_f_/2 cycles because a few cycles are required from the 70% load drop point to reach the final failure. The plastic strain amplitude, Δε_p_/2 was determined from a stress-strain hysteresis loop as specified in BS 7270 (or ISO/DIS) [16], which is defined as the distance between the intersections with the strain axis at the stress-strain hysteresis loop. For each test condition, two to four specimens were used on account of inherent scatter. The fatigue life for each specimen was recorded and the average value was regarded as the fatigue life.

## 3. Results and Discussion

### 3.1. Tensile Test Result

#### 3.1.1. Test by Displacement Control

The mechanical properties of type 316L stainless steel were obtained from tensile tests using the displacement control of 2 mm/min at each test temperature. The elastic modulus was determined by measuring the slope of stress-strain curve during unloading in the elastic region and the yield stress was done by the 0.2% offset method. 

**Figure 2:**
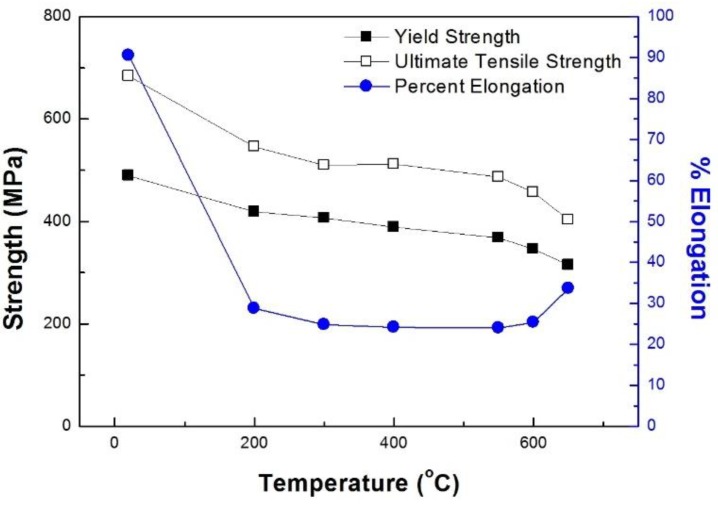
Material properties depending on temperature.

The mechanical properties of type 316L stainless steel are summarized in Table 2. In Figure 2, the material strength (σ_y_, σ_u_) against temperature is plotted, and the ductility change is superimposed to clarify the effect of temperature. As expected, the material strengths and ductility are affected by temperature. The remarkable decrease in strength is observed in the temperature range between RT and 300 °C (16% and 25% decrease in σ_y_ and σ_u_, respectively) and between 550 and 650 °C (17% decrease in both σ_y_ and σ_u_). However, it appears that the temperature effect is not pronounced between 300 and 550 °C. Instead, the material strength at 400 °C is slightly greater than that at 300 °C. Likewise, the material ductility does not change significantly between 300 and 550 °C. However, it decreased significantly between RT and 200 °C and exhibits a minimum value at approximately 400 °C and increases when temperature exceeds 550 °C.

#### 3.1.2. Test by Strain Control

**Figure 3:**
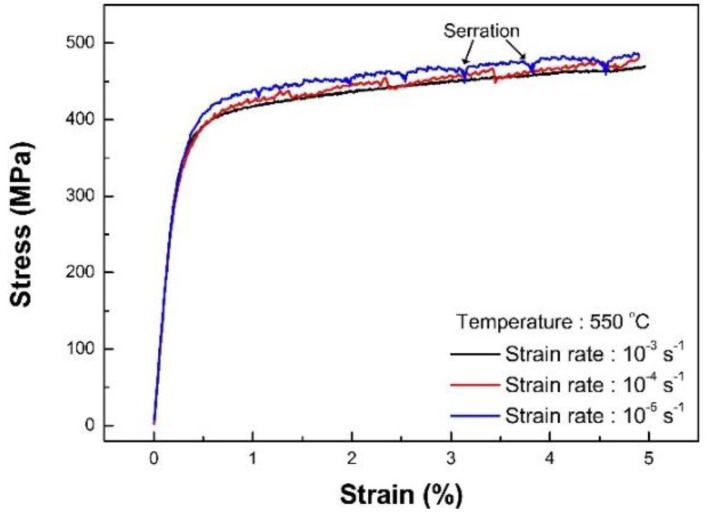
Strain rate dependency at 550 °C.

Strain-controlled tensile test data were provided up to a strain of 5% because the tensile test was deliberately halted, as mentioned in Section 2. The tensile test results at 550 °C using strain rates of 1 × 10^−3^, 1 × 10^−4^ and 1 × 10^−5^ s^−1^ are shown in Figure 3. It is noteworthy that typical engineering metallic materials are generally strengthened at a higher rate of loading and is called “strain-rate hardening” [17]. However, interesting facts were observed in the strain-controlled tensile tests of type 316L stainless steel. In contrast with typical engineering metals, type 316L stainless steel hardens at lower applied strain rates at 550 °C and is known as “a negative strain rate response” [18]. In addition, at small strain rates such as 10^−4^ and 10^−5^ s^−1^, the tensile curve is serrated during plastic deformation and is known as “the plastic serration phenomena.” Therefore, the strain rate affected the material behavior at high temperature and it is speculated that complexities may occur at temperatures from 300 to 550 °C.

According to previous studies [18,19], such abnormal characteristics in the deformation behavior and mechanical properties of type 316L stainless steel at 300–550 °C arise from the interaction between mobile dislocations and solute atoms during plastic deformation, that is, dynamic strain aging (DSA). DSA is known to impair the fatigue property by way of multiple crack initiation, which comes from the DSA-induced localization of deformation, and rapid crack growth due to the DSA-induced hardening.

### 3.2. LCF Test Result

#### 3.2.1. Effect of Temperature on Fatigue Life

LCF tests using a strain control of 1 × 10^−3^ s^−1^ were carried out for strain amplitudes of 0.3% to 0.8% at RT, 200, 400, 550, and 650 °C. It has long been known that the plastic strain amplitude or dissipated energy during a stabilized cycle can be a representative parameter to characterize LCF life. 

**Figure 4:**
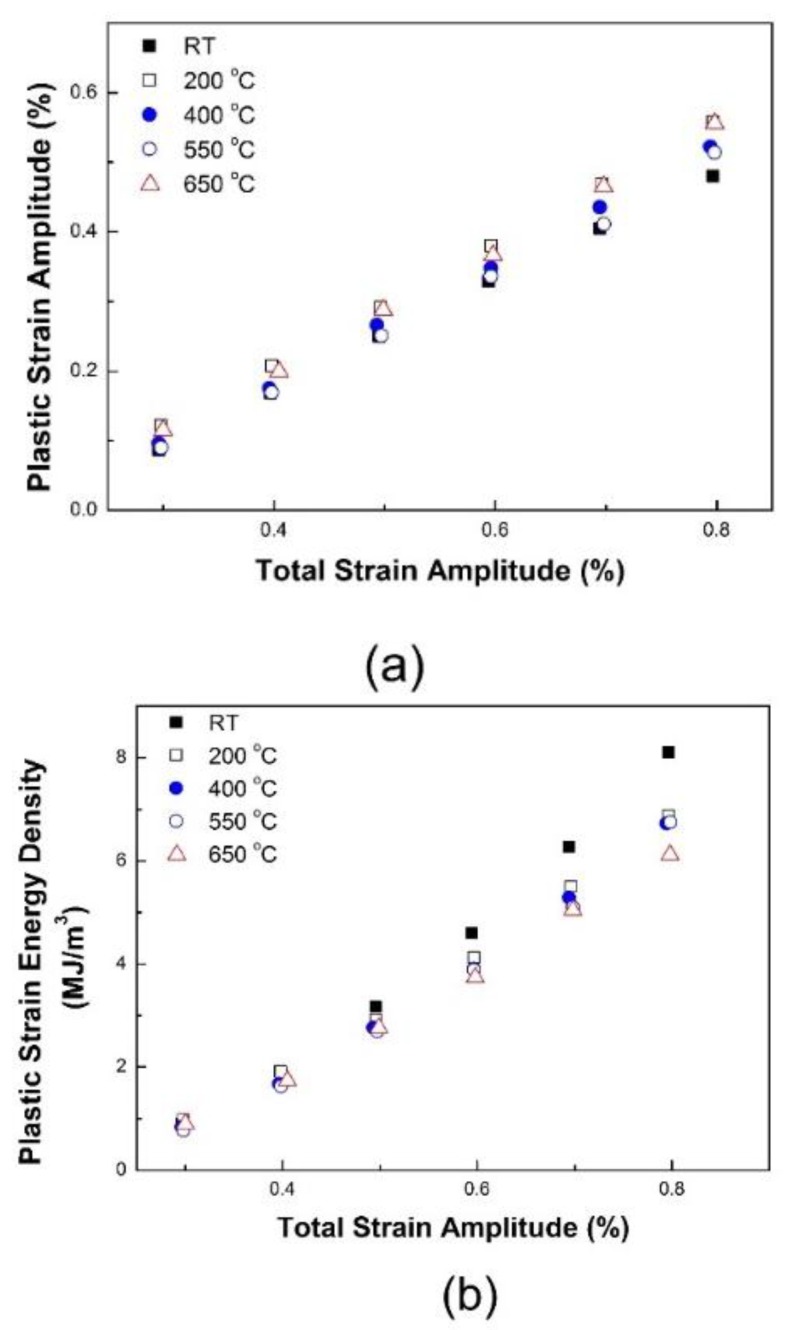
Temperature dependency of (**a**) Δε_p_ and (**b**) ΔW_p_ at $\dot{\varepsilon }=1\times 10^{-3}$ s^−1^.

Figure 4 presents the plastic strain amplitude and plastic strain energy density at a stabilized cycle. Despite the total strain-controlled tests, the tests above are associated with temperature. Thus, tests were performed to determine whether those parameters can be applied correctly regardless of the operating temperature.

**Figure 5:**
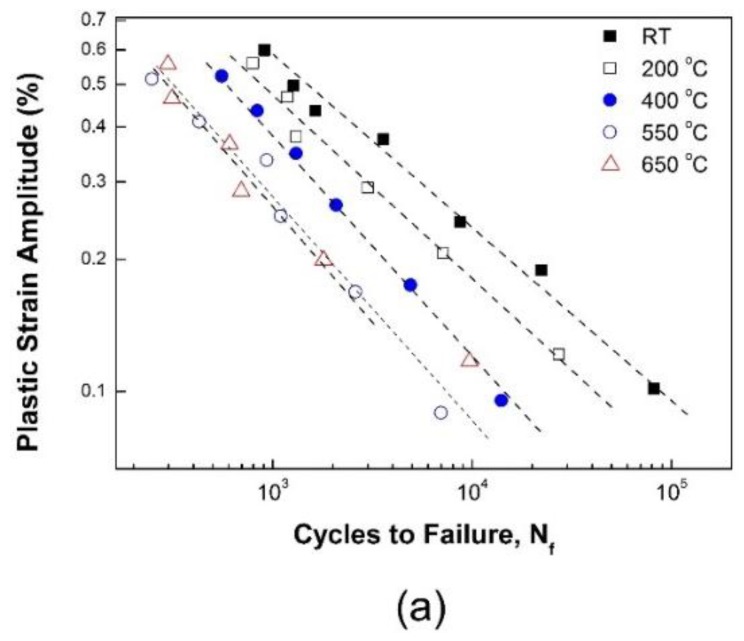

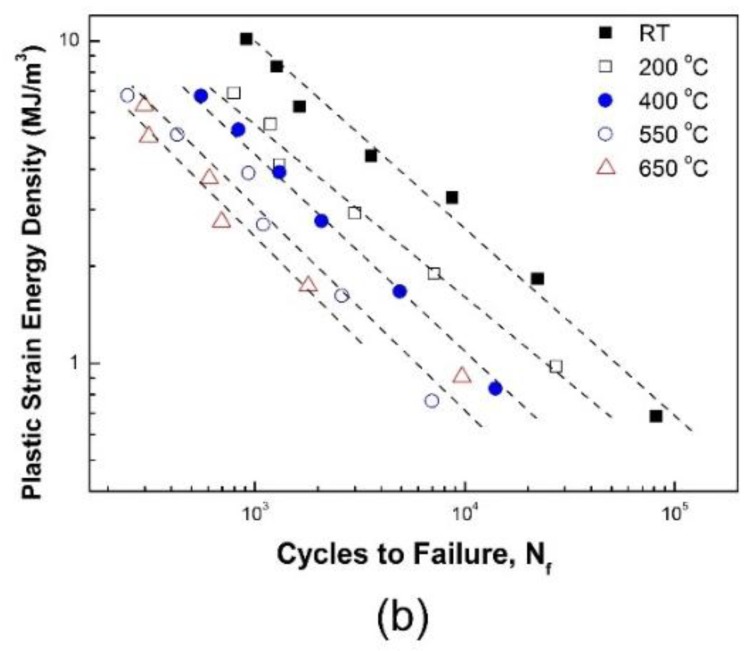
Life prediction by (**a**) the Coffin-Manson model and (**b**) the Morrow model at $\dot{\varepsilon }=1\times 10^{-3}$ s^−1^.

First, the relationship between plastic strain amplitude and fatigue life is shown in Figure 5a. The Coffin-Manson model, in which damage accumulates as the plastic strain cycle is repeated, predicts the fatigue life N_f_ in terms of the plastic strain amplitude Δε_p_/2, as shown in Equation (1):

(1) $$N_{f}^{m_{C}}\left(\Delta \varepsilon_{P}/2\right)=C_{C}$$

where *m*_C_ and *C*_C_ are the fatigue ductility exponent and fatigue ductility coefficient, respectively. As shown in Figure 5a, fatigue life exhibits a linear relationship with the plastic strain amplitude in the logarithmic coordinate at each temperature. The Coffin-Manson model is a good prediction model at each temperature condition, presumably, despite the occurrence of microstructural changes with increased temperature.

Next, the fatigue lives were plotted using the plastic strain energy density in Figure 5b. In this case, the Morrow model was examined, and the model predicts the fatigue life N_f_ in terms of the plastic strain energy density ΔW_p_, as shown in Equation (2):

(2) $$N_{f}^{m_{E}}\Delta W_{P}=C_{E}$$

Here, *m*_E_ and *C*_E_ are material constants determined empirically. The plastic strain energy density was determined from the area enclosed by the stabilized stress-strain hysteresis loop at N_f_/2. As shown in Figure 5b, the plastic strain energy density and fatigue life exhibit a linear relationship in the logarithmic coordinate for each temperature. Likewise, the Morrow model is as a good prediction model for isothermal conditions as is the Coffin-Manson model. 

For simple calculations, the plastic strain energy density of a Masing material is given by

(3) $$\Delta W_{P}=\frac{1-n}{1+n}\Delta \sigma \cdot \Delta \varepsilon_{P},$$

where *n* and Δσ are the cyclic strain hardening exponent in cyclic stress-strain equation and the stress range determined from the stabilized stress-strain hysteresis loop at N_f_/2, respectively. On the other hand, the plastic strain energy density of a non-Masing material can be obtained from the cyclic and master curve equations:

(4) $$\Delta W_{P}=\frac{1-n^{*}}{1+n^{*}}{\Delta \sigma \times \Delta \varepsilon }_{P}+\frac{2n^{*}}{1+n^{*}}{\delta \sigma }_{0}{\times \Delta \varepsilon }_{P},$$

Here, n* is the strain-hardening exponent of the master curve and δσ_0_ is the increase in the proportional stress limit in the master and cyclic curves [20]. 

Both life prediction models, Coffin-Manson and Morrow, are excellent life prediction models for a given temperature condition; however, they cannot be applied irrespective of the temperature. The material constants, *m*_C_, *C*_C_, *m*_E_, and *C*_E_, are summarized in Table 3.

#### 3.2.2. Effect of Strain Rate on Fatigue Life

The effect of strain rate on material behavior and fatigue life is negligible at RT, but it is not negligible as temperature increases. To investigate the effect of strain rate on fatigue life, LCF tests were conducted with diverse strain rates of 1 × 10^−2^, 1 × 10^−3^, 1 × 10^−4^ and 3 × 10^−5^ s^−1^. In those tests, one total strain amplitude of 0.5% was applied and three test temperature conditions, i.e., 400, 550, and 650 °C, were considered. 

The plastic strain energy density obtained from a stabilized hysteresis loop are summarized in Table 4. Depending on the imposed strain rate, the plastic strain energy, *W*_P_, exhibits slight variations. 

With increased strain rate, approximately 10% difference of *W*_P_ occurs between the strain rates of 1 × 10^−2^ and 1 × 10^−4^ s^−1^.

**Figure 6:**
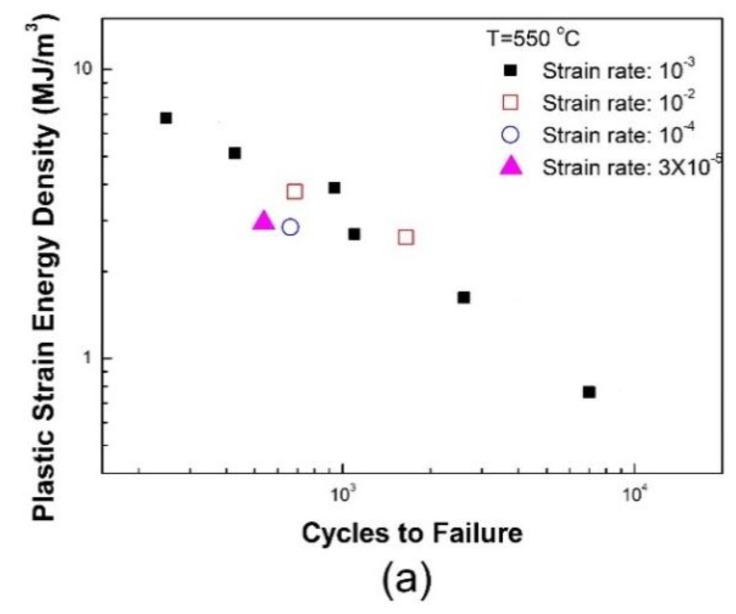

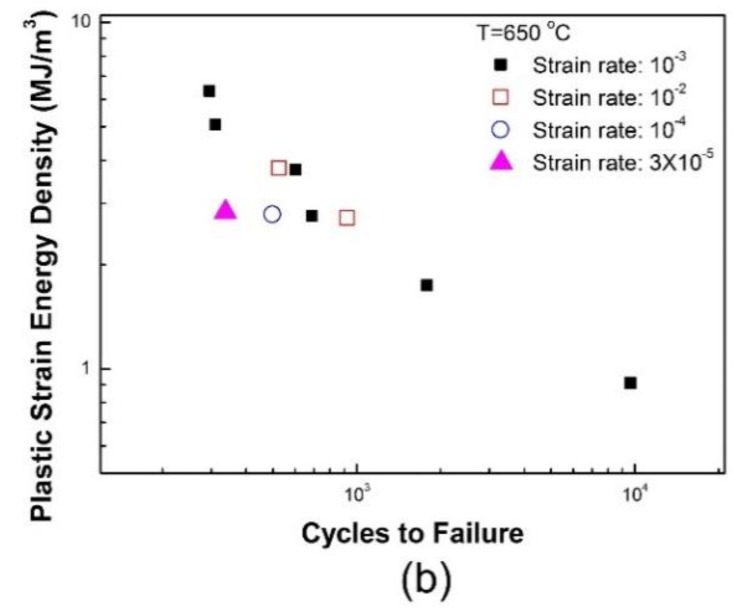
Strain rate dependence on fatigue lives at (**a**) 550 °C and (**b**) 650 °C.

As shown in Figure 6, greater strain rates result in longer fatigue lives. This can be explained based on the DSA effect. DSA is known to degrade the fatigue resistance [18,19]. The DSA-induced hardening accelerates the crack growth rate, consequently reducing the crack propagation life. In the regime of DSA (300–550 °C), the DSA effect becomes more pronounced with a decrease in strain rate and this therefore results in a reduction of fatigue life with decreasing strain rate at a given temperature.

#### 3.2.3. Proposed Energy-Based Fatigue Model

A new life prediction model is developed for a range of temperature and strain rate. It is noteworthy that the material ductility coefficient, *C*_E_, depend significantly on temperature. Further, the dependence of the fatigue exponent, *m*_E_, exists, but not as much as that of *C*_E_ (Table 3). To incorporate temperature dependency into a life prediction model, the temperature-dependent parameters must be properly introduced into the Morrow model to eliminate the temperature dependence of the constants, *m*_E_ and *C*_E_. Thus, a possible model should be of the following form:

(5) $$N_{f}^{m_{E}}\frac{\Delta W_{P}}{f(T)}=C_{E}$$

where *f (T)* is a temperature-dependent normalizing function for ΔW_P_. 

To account for the effect of strain rate on fatigue life, the frequency-modified fatigue life was introduced into Equation (5) by replacing the loading frequency ν by the strain rate $\;\dot{\varepsilon }$ because the strain rate was controlled in the LCF tests. Therefore, the strain-rate inclusive model becomes a multiplicative form of the strain-rate modified fatigue life and normalized dissipate energy density.

(6) $${\left[N_{f}^{}{\left(\frac{\;\dot{\varepsilon }}{{\;\dot{\varepsilon }}_{0}}\right)}^{k-1}\right]}^{m}\frac{\Delta W_{P}}{f(T)}=C$$

Here, ${\dot{\varepsilon }}_{0}$ is a reference strain rate (e.g., 1 × 10^−3^ s^−1^) and *m*, *k*, *C* are material constants. For a proper selection of *f(T)*, the function must be a material parameter that decreases with increasing temperature such as yield stress, material toughness, tangent modulus, etc. Simultaneously, it is reasonable to consider the function as an energy-related function to maintain consistency. 

**Figure 7:**
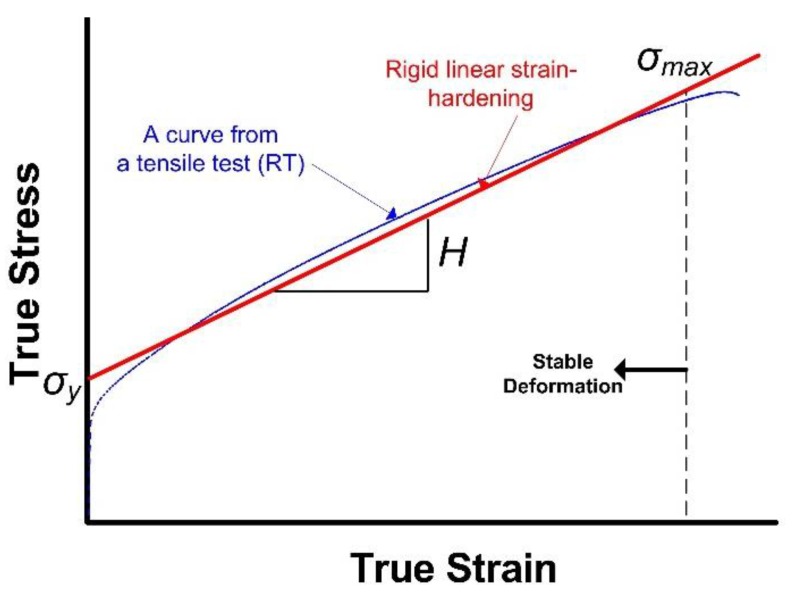
Rigid linear-strain hardening assumption of tensile response.

We begin with a material response to decide the function *f(T)*. As shown in the stress-strain curve presented in Figure 7, the material exhibits a strain-hardening in the plastic deformation region. 

Assuming a rigid-linear strain hardening material, the energy absorbed prior to unstable necking is given by

(7) $$f(T):W=\left(\frac{\sigma_{\max}-\sigma_{Y}}{H}\right)\cdot \left(\frac{\sigma_{\max}+\sigma_{Y}}{2}\right),$$

where *H* and σ_max_ are the tangent modulus and ultimate tensile stress in the true stress-strain curve. However, the energy-related parameter above is not practical for the description of *f(T)*, as shown in Equation (7) because of the number of material constants. For simplification, using a single dominant material constant, $\sigma_{\max}^{2}$, in place of *f(T)*, Equation (6) becomes

(8) $${\left[N_{f}^{}{\left(\frac{\dot{\varepsilon }}{{\dot{\varepsilon }}_{0}}\right)}^{k-1}\right]}^{m}\frac{\Delta W_{P}}{{\left(\sigma_{\max}(T)\right)}^{2}}=C$$

**Figure 8:**
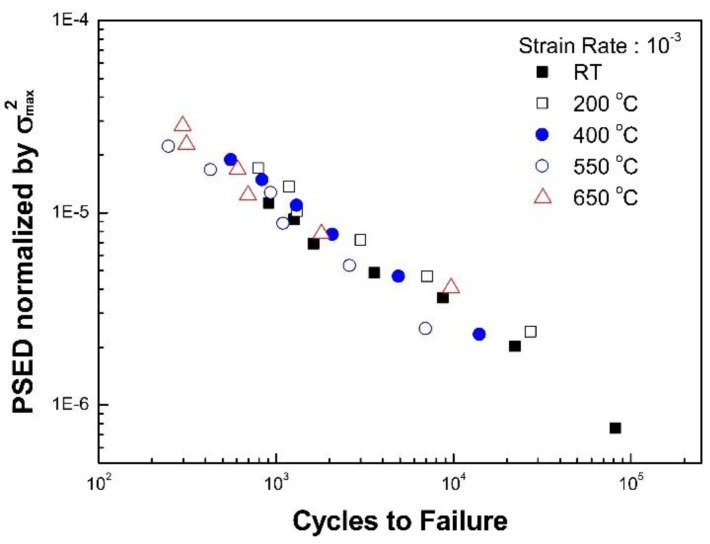
The verification of the new LCF life prediction model with respect to temperature.

When the fatigue life is presented by a function of the plastic strain energy density normalized by $\sigma_{\max}^{2}$, the fatigue life data at the temperature range (RT to 650 °C) was reduced to a single curve as shown in Figure 8. That is, the normalizing factor, $\sigma_{\max}^{2}$, can be employed to eliminate the temperature dependence of the constant C efficiently. 

**Figure 9:**
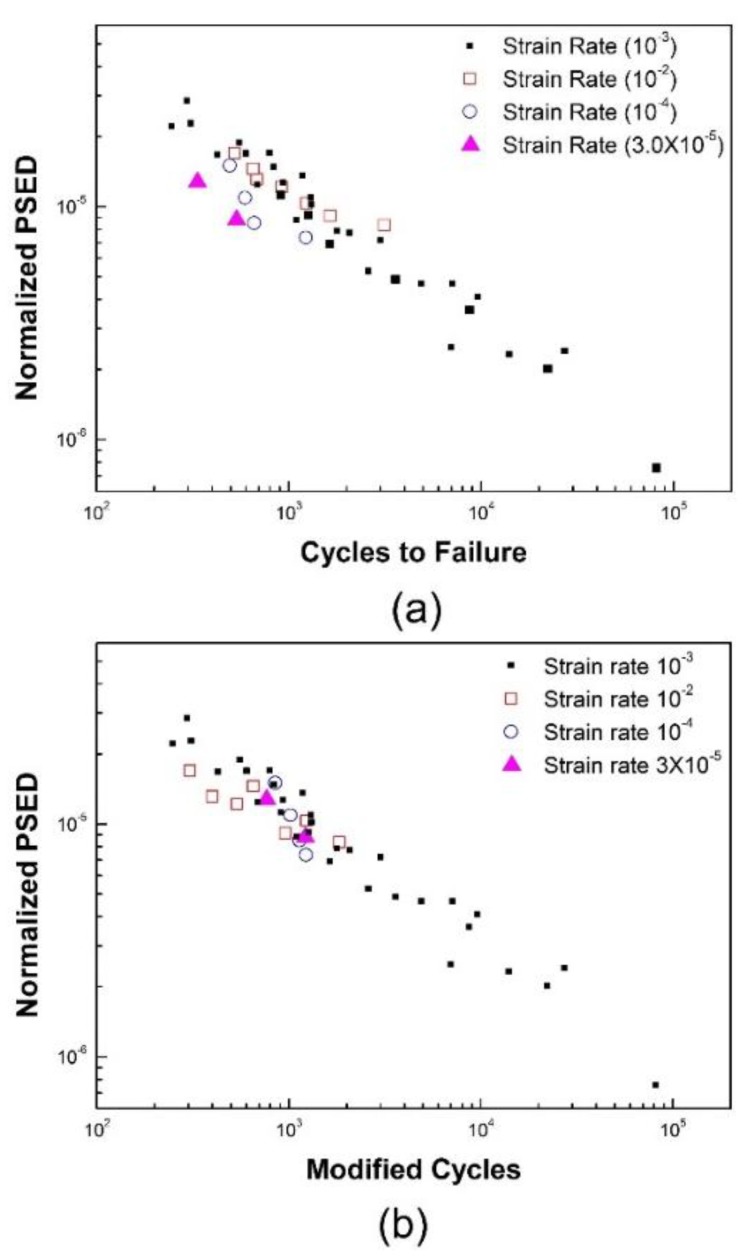
The verification of the new LCF life prediction model with respect to strain-rate: (**a**) without using the strain-rate modified fatigue lives; (**b**) with using strain-rate modified fatigue lives in Equation (8).

The effects of strain rate on fatigue life are examined from Figure 9. Figure 9a does not account for the strain rate dependency on fatigue life, which is based on Equation (6). Meanwhile, Figure 9b is generated by considering the strain rate using Equation (8). When the strain rate modified fatigue life is presented as a function of the temperature modified plastic strain energy density, the data points obtained at the range of temperature (RT to 650 °C) and strain rate (3 × 10^−5^ to 1 × 10^−2^ s^−1^) were reduced into a single curve, as shown in Figure 9b. In this case, the average values of constants m, k, and C in Equation (8) were found to be 0.576, 0.808, and 6.21 × 10^−4^, respectively. 

**Figure 10:**
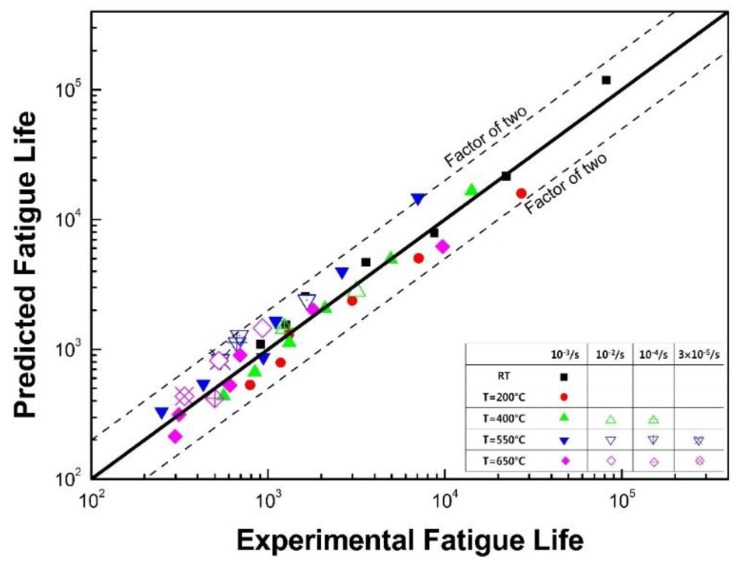
Comparison between predicted fatigue life and test data.

Comparisons between the predicted lives and test data are presented in Figure 10. The horizontal and vertical axes represent the test data and predicted data, respectively. The more data points closer to the line of 45° slope (solid line in the figure) implies that the test results agree well with the predicted lives from the developed model. From the comparison, most of the data points are located between the factor-of-two line (dotted lines in the figure). Therefore, the new life prediction model described in Equation (8) can successfully describe the fatigue life with the inclusion of the effects of strain rate and temperature.

## 4. Conclusions

In this study, an energy-based fatigue life prediction model was developed that included the effects of temperature and strain rate. Tensile behavior of type 316L stainless steel were investigated by varied temperatures and strain rates. Low-cycle fatigue tests were performed over a wide range of temperature (RT to 650 °C) and strain rate (1 × 10^−5^ to 1 × 10^−2^ s^−1^) with a constant total strain amplitude from 0.3% to 0.8%. Fatigue lives obtained from the extensive experimental program were plotted with respect to plastic strain amplitude, plastic strain energy density, and normalized plastic strain energy density. The conclusions were as follows.

1. The developed fatigue model successfully described the temperature and strain-dependent fatigue life. The developed model was a multiplicative form of the normalized plastic strain energy density and frequency modified fatigue life, resulting from a good correlation between the two parameters. Finally, the prediction capabilities were verified by comparing with the predicted life and experimental fatigue life. Consequently, most of experimental data were found to be between the factor-of-two lines.
2. The conventional Coffin-Manson model and Morrow model were recommended at one isothermal temperature for a life prediction model. However, they were not applicable over the specified temperature range, or, the fatigue ductility coefficient, C, and exponent, m, must be provided with respect to temperature.
3. At the temperature range of 300—550 °C, DSA occurred, causing abnormal features in the deformation behavior (a serrated flow and negative strain-rate sensitivity) and mechanical properties (a plateau in the variation of strength and ductility with temperature), and this led to the deterioration of the fatigue resistance. Despite the DSA-induced complicated nature of fatigue life behavior, the capability of the developed fatigue model could be proven over a range of temperature and strain rate.

## Figures and Tables

**Table 1:** The chemical composition of 316L stainless steel (wt %).

C	Si	Mn	P	S	Ni	Cr	Mo	Cu	N
0.025	0.41	1.41	0.025	0.025	10.22	16.16	2.09	–	0.043

**Table 2:** The mechanical properties of 316L stainless steel.

Temperature (°C)	Strain Rate (s^−1^)	σ_y_ (MPa)	σ_u_ (MPa)	EL (%)	σ_max_ (MPa)
20	9.34 × 10^−4^	489	684	50.90	985
200	1.01 × 10^−3^	419	546	28.80	638
300	1.01 × 10^−3^	409	510	24.80	583
400	9.11 × 10^−4^	389	512	24.14	591
550	9.42 × 10^−4^	368	487	24.00	554
600	9.09 × 10^−4^	346	457	25.32	528
650	9.68 × 10^−4^	315	403	33.65	472

**Table 3:** Material constants in each life prediction models.

Temperature (°C)	Coffin-Manson Model (Equation (1))		Morrow Model (Equation (2))	
	*m*_C_	*C*_C_	*m*_E_	*C*_E_
RT	0.395	8.95	0.580	552.33
200	0.418	8.51	0.535	221.38
400	0.501	12.20	0.610	301.30
550	0.510	9.38	0.635	247.07
650	0.526	9.96	0.651	221.31

**Table 4:** The changes of plastic strain energy density, W_P_, with respect to strain rate at each temperature (Δε_t_ = ±0.5%).

Strain Rate (s^−1^)	*W*_P_ (MJ/m^3^)		
	400 °C	500 °C	650 °C
1 × 10^−2^	2.768	2.610	2.701
1 × 10^−3^	2.748	2.662	2.739
1 × 10^−4^	2.828	2.825	2.780
3.2 × 10^−5^	–	2.952	2.837
